# Editorial

**Published:** 1838-02-28

**Authors:** 


### MEDICAL EXAMINER.

PHILADELPHIA.

Wednesday, February 28, 1838.

Our readers, we presume, understand our position in relation to the clinical lectures furnished in our columns. We of course endorse no opinions expressed therein. We have undertaken to present "reports of clinical lectures delivered in the hospitals of Philadelphia;" but with the lecturers remains the responsibility of whatever they have said. Gross personalities we can never admit: we will not consent to be made the means of gratifying individual malice, or to become the organs of private griefs. Remarks, too, uttered in the warmth of an extemporaneous discourse, that we think may be provocative of professional bickerings, it is our desire to omit. On this point, however, we can only advise and consult with the lecturer himself. If he adhere, after reflection, to what he has orally delivered, and disagree with us as to the impropriety of giving it publicity, a sense of editorial independence will not permit us to exclude it. But we disclaim any further identity with our clinical lectures, than such as reports of legislative proceedings attach to a political journal.

